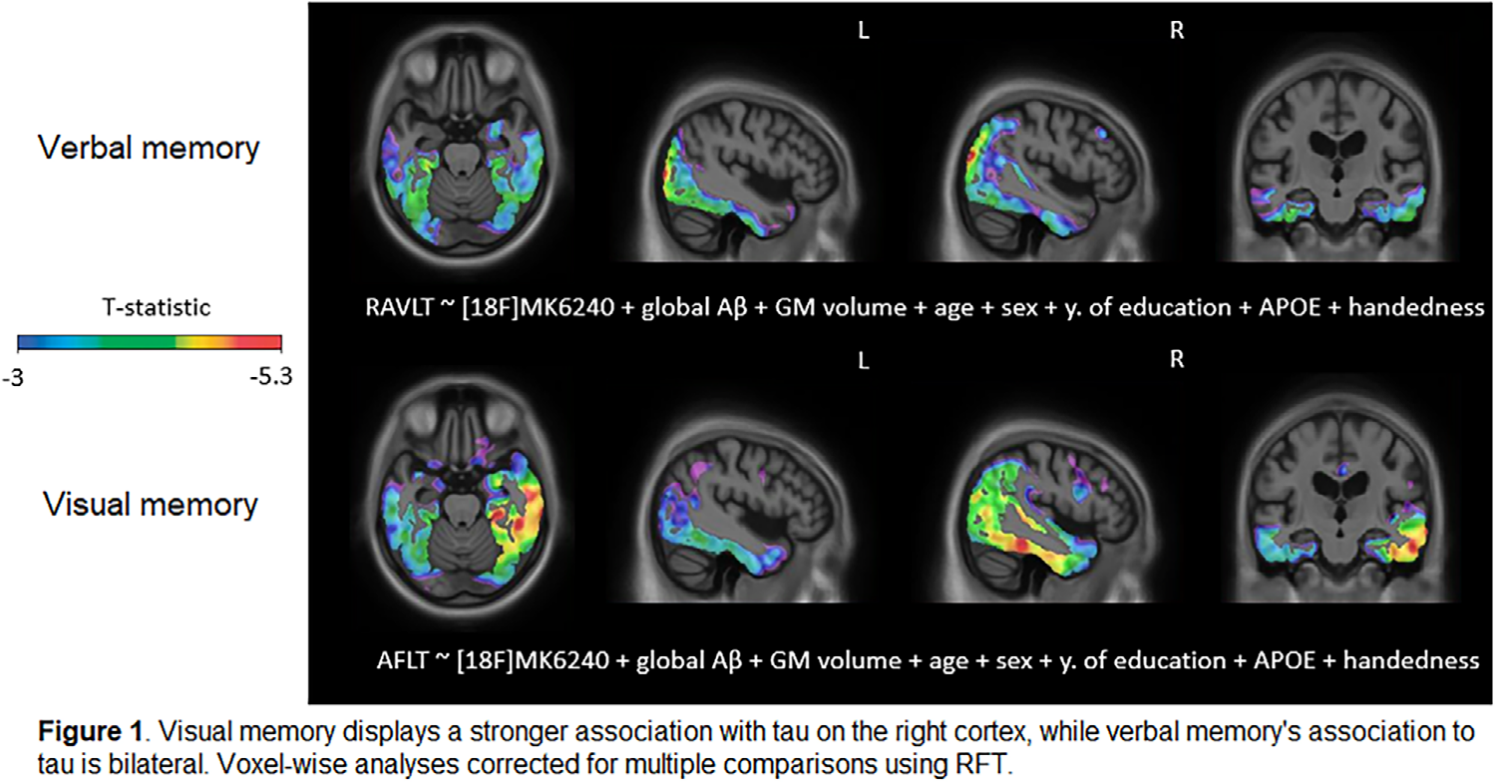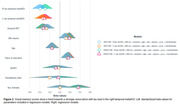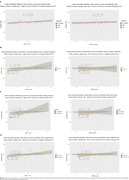# Visual memory is primarily impacted by tau load in the right hemisphere

**DOI:** 10.1002/alz.092784

**Published:** 2025-01-03

**Authors:** Jaime Fernandez Arias, Joseph Therriault, Stijn Servaes, Arthur C. Macedo, Yi‐Ting Wang, Nesrine Rahmouni, Kely Monica Quispialaya Socualaya, Etienne Aumont, Lydia Trudel, Seyyed Ali Hosseini, Brandon J Hall, Tevy Chan, Peter Kunach, Sulantha Mathotaarachchi, Paolo Vitali, Pedro Rosa‐Neto

**Affiliations:** ^1^ McGill University, Montreal, QC Canada; ^2^ Translational Neuroimaging Laboratory, The McGill University Research Centre for Studies in Aging, Montréal, QC Canada; ^3^ Montreal Neurological Institute, Montreal, QC Canada; ^4^ The McGill University Research Centre for Studies in Aging, Montreal, QC Canada; ^5^ Translational Neuroimaging Laboratory, The McGill University Research Centre for Studies in Aging, Montreal, QC Canada; ^6^ Université du Québec à Montréal, Montréal, QC Canada; ^7^ UTSouthwestern, Dallas, TX USA; ^8^ Douglas Mental Health University Institute, Montreal, QC Canada; ^9^ McGill University Research Centre for Studies in Aging, Montreal, QC Canada

## Abstract

**Background:**

Classical literature has pointed at lateralization of the relationship between memory scores and cerebral hemisphere injury. Epilepsy studies have suggested an association between left hippocampal damage and verbal memory deficits, and between right hippocampal damage and visual memory deficits. We aimed to explore this concept in the context of tauopathy due to Alzheimer’s disease.

**Method:**

we assessed 132 cognitively unimpaired (CU) older adults, and 44 cognitively impaired (CI), amyloid‐β positive individuals from the Translational Biomarkers in Aging and Dementia (TRIAD) cohort. All participants had amyloid‐PET with [18F]AZD4694 and tau‐PET with [18F]MK6240, verbal memory assessments with Rey Auditory Verbal Learning Test (RAVLT), and visual memory assessments with Aggie Figures Learning Test (AFLT). We conducted voxel‐wise analyses to explore the relationship between tau PET and memory modality. In addition, we run regression models with right or left tau temporal metaROI as predictor and memory scores as outcome variables. We entered global amyloid (PET), sex, age, years of education, ApoE status, grey matter volume and handedness as covariates. Finally, we used the laterality index (LI) to investigate correlations with memory modality. Right laterality is indicated by an LI below 0, while left laterality would be signaled by an LI above 0.

**Result:**

voxel‐wise analyses suggested a lateralized relationship between tau and visual memory scores on the right hemisphere, whereas tau and verbal memory scores were bilaterally associated. Regression analyses showed a trend towards a stronger relationship between temporal tau on the right and visual memory scores. The LI was significantly associated with visual memory performance. Further analyses showed that this relationship seems to be driven by tau positive individuals.

**Conclusion:**

visual memory performance might be more prominently affected by tau load on the right hemisphere in Alzheimer’s disease. Furthermore, a hemispheric imbalance in the amount of tau is related to visual, but not verbal, memory scores.